# A Lattice-Theoretic Approach to Multigranulation Approximation Space

**DOI:** 10.1155/2014/785932

**Published:** 2014-08-27

**Authors:** Xiaoli He, Yanhong She

**Affiliations:** College of Science, Xi'an Shiyou University, Xi'an 710065, China

## Abstract

In this paper, we mainly investigate the equivalence between multigranulation approximation space and single-granulation approximation space from the lattice-theoretic viewpoint. It is proved that multigranulation approximation space is equivalent to single-granulation approximation space if and only if the pair of multigranulation rough approximation operators $\left(\bar{\Sigma_{i=1}^{n}R_{i}},\underline{\Sigma_{i=1}^{n}R_{i}}\right)$ forms an order-preserving Galois connection, if and only if the collection of lower (resp., upper) definable sets forms an (resp., union) intersection structure, if and only if the collection of multigranulation upper (lower) definable sets forms a distributive lattice when *n* = 2, and if and only if $\forall X\subseteq U,\underline{\Sigma_{i=1}^{n}R_{i}}(X)=\underline{\cap_{i=1}^{n}R_{i}}(X)$. The obtained results help us gain more insights into the mathematical structure of multigranulation approximation spaces.

## 1. Introduction

The theory of rough sets, proposed by Pawlak [1, 2], is a formal tool for the study of intelligent systems characterized by insufficient and incomplete information. After over thirty years of progress, it has become a well-established mechanism for uncertainty management in a wide variety of applications related to artificial intelligence (see [3, 4]).

Pawlak's rough set theory is defined on the basis of an approximation space (*U*, *R*), where *U* is a nonempty set, also called the universe of discourse, and *R* is an equivalence relation on *U*, representing the indiscernibility at the object level due to the lack of knowledge or information. Owing to the indiscernibility of objects, some subsets of the universe cannot be completely characterized with the available knowledge, thus forming a region of uncertainty. Pawlak's idea was to approximate those sets with two precise sets from below and above based on certainty and possibility, respectively. They are called lower and upper approximations and are defined, for any *X*⊆*U*, as

(1) R_(X)={x∈U ∣ [x]R⊆X},R¯(X)={x∈U ∣ [x]R∩X≠∅}.

Since in Pawlak's rough set theory, the concept is depicted by known knowledge induced from a single equivalence relation on the universe, in view of granular computing (see [5, 6]), Pawlak's rough set theory was established through a single granulation, and therefore (*U*, *R*) is also called a single-granulation approximation space. However, as illustrated in [7], in some cases it is more reasonable to describe the target concept through multiple relations on the universe according to user requirements or targets of problem solving. To more widely apply the rough set theory in practical applications, Qian et al. extended Pawlak's single-granulation rough set model to a multigranulation rough set model (see [7]). To date, the theory of multigranulation rough set progressed rapidly. Many interesting results have been reported in the literature ([8–15]).

Superficially, the notion of multigranulation rough set differs significantly from that of Pawlak's single-granulation rough set, because the former is defined by using multiple equivalence relations on the universe whereas the latter is defined by employing a single one. However, in some situations (see Example 5), the collection of multigranulation rough sets generated by a multigranulation approximation space may coincide with that produced by a single-granulation approximation space, such a phenomenon will be referred to as equivalence between these two kinds of approximation spaces. Since the lattice-theoretic properties of Pawlak's single-granulation rough sets have been extensively studied, then the equivalence between these two kinds of approximation spaces will help us gain more insights into the mathematical structure of multigranulation rough sets and hence deserves further study. Motivated by the above considerations, we attempt to investigate the conditions under which the multigranulation approximation space is equivalent to a single-granulation approximation space from the lattice-theoretic viewpoint.

The rest of the paper proceeds as follows: we briefly review in Section 2 multigranulation rough set theory and some of its basic properties. In Section 3, we present the main results and give their detailed proof. In Section 4, we complete this paper with some concluding remarks.

## 2. Preliminary

A multigranulation approximation space [7] is a pair (*U*, {*R*_*i*_}_*i*=1_^*n*^), where *U* is a nonempty set, also called the universe of discourse, and each *R*_*i*_ (1 ≤ *i* ≤ *n*) is an equivalence relation on *U*, representing a particular kind of indiscernibility at the level of objects. For *X*⊆*U*, define

(2) Σi=1nRi_(X)={x:[x]R1⊆X,or  [x]R2 ⊆X,…,or  [x]Rn⊆X},

(3) Σi=1nRi¯(X) ={x:[x]R1∩X≠∅,[x]R2∩X≠∅,…,   [x]Rn∩X≠∅}.

Then we call $\underline{\Sigma_{i=1}^{n}R_{i}}(X)$, $\bar{\Sigma_{i=1}^{n}R_{i}}(X)$ the multigranulation lower approximation and the multigranulation upper approximation of *X*, respectively. Note that, in [7], two kinds of multigranulation rough approximations were defined, they are optimistic multigranulation rough approximation and pessimistic rough multigranulation approximation. The above defined $\bar{\Sigma_{i=1}^{n}R_{i}}$, $\underline{\Sigma_{i=1}^{n}R_{i}}$ are actually the optimistic rough approximation operators. However, since we are not concerned with the pessimistic one in the present paper, the term multigranulation approximation always means the optimistic multigranulation approximation. If $\underline{\Sigma_{i=1}^{n}R_{i}}(X)=X$, then we call *X* a multigranulation lower definable set, if $\bar{\Sigma_{i=1}^{n}R_{i}}(X)=X$, then we call *X* a multigranulation upper definable set and if $\underline{\Sigma_{i=1}^{n}R_{i}}(X)=X=\bar{\Sigma_{i=1}^{n}R_{i}}(X)$, then we call *X* a multigranulation definable set. Let *x* ∈ *U*, if there exists an equivalence relation *R*_*k*_ ∈ {*R*_1_,…, *R*_*n*_} such that [*x*]_*R*_*k*__ = ∩_*i*=1_^*n*^[*x*]_*R*_*i*__; that is, [*x*]_*R*_*k*__ is the smallest equivalence class containing *x*; then we say that *x* has the SEC property.

**Proposition 1 (see [1]).**

Let (*U*, *R*) be a single-granulation approximation space. Then the collection of definable sets in (*U*, *R*) forms a Boolean algebra under the usual set-theoretic operations.

**Definition 2 (see [15]).**

The multigranulation approximation space (*U*, {*R*_*i*_}_*i*=1_^*n*^) is said to be equivalent to a single-granulation approximation space (*U*, *R*), if ∀*X*⊆*U*, $\underline{\Sigma_{i=1}^{n}R_{i}}(X)=\underline{R}(X)$ and $\bar{\Sigma_{i=1}^{n}R_{i}}(X)=\bar{R}(X)$.

**Proposition 3 (see [15]).**

Let (*U*, {*R*_*i*_}_*i*=1_^*n*^) be a multigranulation approximation space. Then (*U*, {*R*_*i*_}_*i*=1_^*n*^) is equivalent to a single-granular approximation space (*U*, *R*) if and only if each element of *U* has the SEC property.

**Proposition 4 (see [15]).**

Let (*U*, {*R*_*i*_}_*i*=1_^*n*^) be a multigranulation approximation space, $\underline{H}=\{\underline{\Sigma_{i=1}^{n}R_{i}}(X)|X\subseteq U\}$, and $\bar{H}=\{\bar{\Sigma_{i=1}^{n}R_{i}}(X)|X\subseteq U\}$. Then $(\underline{H},\vee ,\wedge ,Ø,U)\;\;($resp., $\left(\bar{H},\vee ,\wedge ,Ø,U)\right)$ forms a bounded lattice with the operations ∨, ∧ defined by $\forall A,B\in \underline{H}$, *A*∨*B* = *A* ∪ *B*, $A\wedge B=\underline{\Sigma_{i=1}^{n}R_{i}}\left(A\cap B\right)$ (resp., $\forall A,B\in \bar{H}$, *A*∧*B* = *A*∩*B*, $A\cup B=\bar{\Sigma_{i=1}^{n}R_{i}}(A\cup B)$).

## 3. Main Results

We begin with an example, which shows that in some situations, the multigranulation approximation space is equivalent to a single-granulation approximation space.

**Example 5 .**

Let *U* = {*a*, *b*, *c*, *d*, *e*, *f*}, *R*_1_ = {{*a*, *b*, *c*}, {*d*, *e*}, {*f*}}, *R*_2_ = {{*a*, *b*}, {*c*}, {*d*, *e*, *f*}}. Then an easy verification shows that the multigranulation approximation space (*U*, {*R*_*i*_}_*i*=1_^2^) is actually equivalent to a single-granulation approximation space (*U*, *R*), where *R* = {{*a*, *b*}, {*c*}, {*d*, *e*}, {*f*}}.

Then one natural question arises: under what conditions the notion of multigranulation approximation space reduce to single-granulation space. Such a consideration leads us to investigate several necessary and sufficient conditions under which multigranulation approximation spaces and single-granulation approximation spaces are equivalent to each other.

Some preliminary results of Galois connections are briefly recalled below.

Let (*P*, ≤), (*Q*, ≤) be two ordered sets and *f* : *P* → *Q*, *g* : *Q* → *P* two mappings. The pair (*f*, *g*) is said to be an order-preserving Galois connection between *P* and *Q* if ∀*p* ∈ *P*, *q* ∈ *Q*, *f*(*p*) ≤ *q*⇔*p* ≤ *g*(*q*). It can be easily checked that both *f* and *g* are order-preserving, and hence we also call (*f*, *g*) an order-preserving Galois connection.

**Proposition 6 (see [16]).**

Let *f* : *P* → *Q*, *g* : *Q* → *P* be two mappings defined on ordered sets *P*, *Q*, respectively. Then (*f*, *g*) forms an order-preserving Galois connection if and only if

- ∀*p* ∈ *P*, *q* ∈ *Q*, *p* ≤ *g* (*f*(*p*)), *q* ≤ *f* (*g*(*q*)),
- both *f* and *g* are order-preserving.

It was shown in [16] that the pair of Pawlak's single-granulation rough approximation operators $\left(\bar{R},\underline{R}\right)$ forms an order-preserving Galois connection.

**Proposition 7 .**

Let (*U*, {*R*_*i*_}_*i*=1_^*n*^), (*U*, *R*) be a multigranulation approximation space and a single-granulation approximation space, respectively. Then (*U*, {*R*_*i*_}_*i*=1_^*n*^) is equivalent to (*U*, *R*) if and only if $\left(\bar{\Sigma_{i=1}^{n}R_{i}},\underline{\Sigma_{i=1}^{n}R_{i}}\right)$ forms an order-preserving Galois connection.

**Proof**

“⇒” According to the definition of equivalence between (*U*, {*R*_*i*_}_*i*=1_^*n*^) and (*U*, *R*), we have $\bar{\Sigma_{i=1}^{n}R_{i}}=\bar{R}$ and $\underline{\Sigma_{i=1}^{n}R_{i}}=\underline{R}$; then, the desired result follows immediately from the fact that $\left(\bar{R},\underline{R}\right)$ forms an order-preserving Galois connection.

“⇐” We suppose, on the contrary, that multigranulation approximation (*U*, {*R*_*i*_}_*i*=1_^*n*^) is not equivalent to any single-granulation approximation space. Then by Proposition 3, there exits one element *a* ∈ *U* such that *a* does not have the SEC property. Take *X* = ∩_*i*=1_^*n*^[*a*]_*R*_*i*__, we will show that $\underline{\Sigma_{i=1}^{n}R_{i}}\;(\bar{\Sigma_{i=1}^{n}R_{i}}(X))=Ø$ below. To this end, we will firstly prove that $\bar{\Sigma_{i=1}^{n}R_{i}}(X)=X$. Considering the fact that $\bar{\Sigma_{i=1}^{n}R_{i}}(X)⊇X$, it suffices to show that ∀*x* ∈ *U*, *x* ∉ *X* implies $x\notin \bar{\Sigma_{i=1}^{n}R_{i}}(X)$.

There are two cases to be considered below.

*Case 1*. ∀*i* ∈ {1,2,…, *m*} such that *x* ∉ [*a*]_*R*_*i*__.

In this case, we have that ∀*i* ∈ {1,2,…, *n*}, [*x*]_*R*_*i*__∩[*a*]_*R*_*i*__ = *Ø*, consequently, [*x*]_*R*_*i*__∩*X* = [*x*]_*R*_*i*__∩∩_*i*=1_^*n*^[*a*]_*R*_*i*__ = *Ø*, whence the result follows immediately.

*Case 2*. Consider ∃*i* ∈ {1,2,…, *n*} such that *x* ∈ [*a*]_*R*_*i*__. Since *x* ∉ *X* = ∩_*i*=1_^*n*^[*a*]_*R*_*i*__, we conclude that there exists at least one equivalence class [*a*]_*R*_*j*__ (*i* ≠ *j*) such that *x* ∉ [*a*]_*R*_*j*__. This shows that [*x*]_*R*_*j*__∩[*a*]_*R*_*j*__ = *Ø*, consequently, [*x*]_*R*_*j*__∩∩_*i*=1_^*n*^[*a*]_*R*_*j*__ ≠ *Ø*, then the desired result follows immediately from (3).

Then, we will further show that $\underline{\Sigma_{i=1}^{n}R_{i}}(X)=Ø$. Suppose, on the contrary, that $\underline{\Sigma_{i=1}^{n}R_{i}}(X)\neq Ø$, that is, there exists *x* ∈ *U* such that $x\in \underline{\Sigma_{i=1}^{n}R_{i}}(X)$. According to (2), there exists *j* ∈ {1,2,…, *n*} such that [*x*]_*R*_*i*__⊆*X* = ∩_*i*=1_^*n*^[*a*]_*R*_*i*__, which means that *x* is contained in each equivalence class containing *a*, consequently, [*x*]_*R*_*j*__ = [*a*]_*R*_*j*__. We can thus conclude from [*a*]_*R*_*i*__⊆∩_*i*=1_^*n*^[*a*]_*R*_*i*__ that [*x*]_*R*_*j*__ is the smallest equivalence class containing *x*, a contradiction.

Since *X* = ∩_*i*=1_^*n*^[*x*]_*R*_*i*__ contains at least one point *x* and hence is a nonempty set. Combining the result proved above, we conclude that $X⊈\underline{\Sigma_{i=1}^{n}R_{i}}(\bar{\Sigma_{i=1}^{n}R_{i}}(X))$, which, however, contradicts with the precondition that $\left(\bar{\Sigma_{i=1}^{n}R_{i}},\underline{\Sigma_{i=1}^{n}R_{i}}\right)$ forms an order-preserving Galois connection, as desired.

Let $\underline{H}=\{\underline{\Sigma_{i=1}^{n}R_{i}}\left(X\right)|X\subseteq U\}$; that is, $\underline{H}$ is the collection of lower approximations of subsets in (*U*, {*R*_*i*_}_*i*=1_^*n*^). The following example shows that $\underline{H}$ does not form an intersection structure (see [17]) in the general case. For instance, let *U* = {*a*, *b*, *c*, *d*, *e*}, *R*_1_ = {{*a*, *b*}, {*c*, *d*}, {*e*}}, and *R*_2_ = {{*a*}, {*b*, *c*}, {*d*, *e*}}. Observe that $\left\{a,b\right\},\left\{b,c\right\}\in \underline{H}$; however, $\{a,b\}\cap \{b,c\}=\{b\}\notin \underline{H}$.

The following proposition provides another necessary and sufficient condition for the equivalence between multigranulation approximation spaces and single-granulation approximation spaces.

**Proposition 8 .**

Let (*U*, {*R*_*i*_}_*i*=1_^*n*^) be a multigranulation approximation space. Then (*U*, {*R*_*i*_}_*i*=1_^*n*^) is equivalent to a single-granular approximation space (*U*, *R*) if and only if $\underline{H}$ forms an intersection structure.

**Proof**

“⇒” It follows immediately from Proposition 1.

“⇐” Suppose, on the contrary, that the multigranulation approximation space is not equivalent to any single-granulation approximation space; then, there exists an element of *U* (say as *a*) such that *a* does not have the SEC property. Since ${\left[a\right]}_{R_{i}}\in \underline{H}$, 1 ≤ *i* ≤ *n*, we then have from the fact that $\underline{H}$ forms an intersection structure that $\cap_{i=1}^{n}{\left[a\right]}_{R_{i}}\in \underline{H}$. Clearly, *a* ∈ ∩_*i*=1_^*n*^[*a*]_*R*_*i*__, and then according to (2), there exists some [*a*]_*R*_*j*__ (1 ≤ *j* ≤ *n*) such that [*a*]_*R*_*j*__⊆∩_*i*=1_^*n*^[*a*]_*R*_*i*__, implying that [*a*]_*R*_*j*__ = ∩_*i*=1_^*n*^[*a*]_*R*_*i*__, which, however, is a contradiction with the fact that *a* does not have the SEC property.

Similarly, we can prove the following result.

**Proposition 9 .**

Let (*U*, {*R*_*i*_}_*i*=1_^*n*^) be a multigranulation approximation space. Then (*U*, {*R*_*i*_}_*i*=1_^*n*^) is equivalent to a single-granular approximation space (*U*, *R*) if and only if $\bar{H}$ forms an union structure.

**Proposition 10 .**

Let (*U*, {*R*_*i*_}_*i*=1_^2^) be a multigranulation approximation space with *n* = 2. Then (*U*, {*R*_*i*_}_*i*=1_^2^) is equivalent to a single-granular approximation space (*U*, *R*) if and only if $\left(\underline{H},\vee ,\wedge ,Ø,U\right)$ is a distributive lattice.

**Proof**

“⇒” It follows immediately from Proposition 1.

“⇐” Suppose, on the contrary, that the multigranulation approximation space (*U*, {*R*_*i*_}_*i*=1_^2^) is not equivalent to any single-granulation approximation space, then according to Proposition 3, there exists an element of *U*, say as *b*, such that [*b*]_*R*_1__⊈[*b*]_*R*_2__ and [*b*]_*R*_2__⊈[*b*]_*R*_1__. Therefore, there exist two elements *a*, *c* satisfying *a* ∈ [*b*]_*R*_1__, *a* ∉ [*b*]_*R*_2__, *c* ∈ [*b*]_*R*_2__, and *c* ∉ [*b*]_*R*_1__. We assume, without any loss of generality, that

(4) R1={…{a,b,b1,…,bn,d1,…,dm}…},R2={…{a,a1,…,as},{b,b1,…,bn,c,e1,…,et}…},

where {*b*, *b*_1_,…, *b*_*n*_} = [*b*]_*R*_1__∩[*b*]_*R*_2__.

There are two cases to be considered below.

*Case 1.* Consider {*d*_1_,…, *d*_*m*_} = *Ø*. Let *X* = {*a*, *b*, *b*_1_,…, *b*_*n*_}, *Y* = {*a*, *a*_1_,…, *a*_*s*_}, *Z* = {*b*, *b*_1_,…, *b*_*n*_, *c*, *e*_1_,…, *e*_*t*_}. Clearly, $X,Y,Z\in \underline{H}$. An easy verification shows that

(5) X∧(Y∨Z) ={a,b,b1,…,bn}  ∧({a,a1,…,as}∨{b,b1,…,bn,c,e1,…,et}) ={a,b,b1,…,bn}  ∧({a,a1,…,as,b,b1,…,bn,c,e1,…,et}) =Σi=1nRi_({a,b,b1,…,bn}   ∩({a,a1,…,as,b,b1,…,bn,c,e1,…,et})) =Σi=1nRi_{a,b,b1,…,bn}={a,b,b1,…,bn},(X∧Y)∨(X∧Z) =({a,b,b1,…,bn}∧{a,a1,…,as})  ∨({a,b,b1,…,bn}∧{b,b1,…,bn,c,e1,…,et}) =Σi=1nRi_({a,b,b1,…,bn}∩{a,a1,…,as})  ∪Σi=1nRi_({a,b,b1,…,bn}∩{b,b1,…,bn,c,e1,…,et}) =Σi=1nRi_({a})∪Σi=1nRi_({b,b1,…,bn}) =Σi=1nRi_({a})∪Ø=Σi=1nRi_({a}).

Since *b* definitely does not belong to $\underline{\Sigma_{i=1}^{n}R_{i}}(\{a\})$, we obtain (*X*∧*Y*)∨(*X*∧*Z*) ≠ *X*∧(*Y*∨*Z*), showing that $\underline{H}$ is not distributive.

*Case 2.* Consider {*d*_1_,…, *d*_*m*_} ≠ *Ø*. In this case, take *X* = {*a*, *b*, *b*_1_,…, *b*_*n*_, *d*_1_,…, *d*_*m*_}, *Y* = [*a*]_*R*_2__ ∪ [*d*_1_]_*R*_2__ ∪ ⋯∪[*d*_*m*_]_*R*_2__, *Z* = {*b*, *b*_1_,…, *b*_*n*_, *c*, *e*_1_,…, *e*_*t*_}. Clearly, $X,Y,Z\in \underline{H}$. An easy verification shows that

(6) (X∧Y)∨(X∧Z) =({a,b,b1,…,bn,d1,…,dm}∧([a]R2∪[d1]R2∪⋯∪[dm]R2))∨({a,b,b1,…,bn,d1,…,dm} ∧{b,b1,…,bn,c,e1,…,et}) =Σi=1nRi_({a,b,b1,…,bn,d1,…,dm} ∩([a]R2∪[d1]R2∪⋯∪[dm]R2))  ∪Σi=1nRi_({a,b,b1,…,bn,d1,…,dm} ∩{b,b1,…,bn,c,e1,…,et}) =Σi=1nRi_({a,d1,…,dm})∪Σi=1nRi_({b,b1,…,bn}) =Σi=1nRi_({a,d1,…,dm}),X∧(Y∨Z) ={a,b,b1,…,bn,d1,…,dm}  ∧(([a]R2∪[d1]R2∪⋯∪[dm]R2)∨{b,b1,…,bn,c,e1,…,et}) ={a,b,b1,…,bn,d1,…,dm}  ∧(([a]2∪[d1]2∪⋯∪[dm]2)∪{b,b1,…,bn,c,e1,…,et}) =Σi=1nRi_({a,b,b1,…,bn,d1,…,dm}∩(([a]R2∪[d1]R2∪⋯∪[dm]R2)∪{b,b1,…,bn,c,e1,…,et})) =Σi=1nRi_({a,b,b1,…,bn,d1,…,dm}) ={a,b,b1,…,bn,d1,…,dm}.

Since *b* definitely does not belong to (*X*∧*Y*)∨(*X*∧*Z*), we obtain (*X*∧*Y*)∨(*X*∧*Z*) ≠ *X*∧(*Y*∨*Z*), showing that $\underline{H}$ is not distributive.

The following proposition can be shown in a similar way.

**Proposition 11 .**

Let (*U*, {*R*_*i*_}_*i*=1_^2^) be a multigranulation approximation space. Then (*U*, {*R*_*i*_}_*i*=1_^2^) is equivalent to a single-granular approximation space (*U*, *R*) if and only if $\left(\bar{H},\vee ,\wedge ,Ø,U\right)$ is a distributive lattice.

In [7], some sufficient and necessary conditions for the equivalence between a multigranulation approximation space and a single-granulation approximation space are provided mainly from the viewpoint of topology, that is, as follows.

**Proposition 12 .**

Let (*U*, {*R*_*i*_}_*i*=1_^*n*^) be a multigranulation approximation space. Then (*U*, {*R*_*i*_}_*i*=1_^*n*^) is equivalent to a single-granular approximation space (*U*, *R*) if and only if $\underline{H}$ forms a topology on *U*.

**Proposition 13 .**

Let (*U*, {*R*_*i*_}_*i*=1_^*n*^) be a multigranulation approximation space. Then (*U*, {*R*_*i*_}_*i*=1_^*n*^) is equivalent to a single-granular approximation space (*U*, *R*) if and only if $\bar{H}$ forms a topology on *U*.

**Proposition 14 .**

Let (*U*, {*R*_*i*_}_*i*=1_^*n*^) be a multigranulation approximation space. Then (*U*, {*R*_*i*_}_*i*=1_^*n*^) is equivalent to a single-granular approximation space (*U*, *R*) if and only if ∀*X*⊆*U*, $\underline{\Sigma_{i=1}^{n}R_{i}}(X)=\underline{\cap_{i=1}^{n}R_{i}}(X)$.

**Proof**

“⇒” If (*U*, {*R*_*i*_}_*i*=1_^*n*^) is equivalent to a single-granular approximation space (*U*, *R*), then according to Definition 2, we have ∀*X*⊆*U*, $\underline{\Sigma_{i=1}^{n}R_{i}}(X)=\underline{R}(X)$. Let *x* ∈ *U*, by taking *X* = [*x*]_*R*_*i*__ (1 ≤ *i* ≤ *n*), one can calculate that $\underline{\Sigma_{i=1}^{n}R_{i}}(X)=\underline{\Sigma_{i=1}^{n}R_{i}}({\left[x\right]}_{R_{i}})={\left[x\right]}_{R_{i}}=\underline{R}({\left[x\right]}_{R_{i}})$, which shows that [*x*]_*R*_*i*__ is a definable set in the single-granulation approximation space (*U*, *R*). Since the collection of definable sets in (*U*, *R*) forms a Boolean algebra and thus is closed under set-theoretic intersection, we conclude that ∩_*i*=1_^*n*^[*x*]_*R*_*i*__ is also a definable set in (*U*, *R*). Moreover, since the collection of ∩_*i*=1_^*n*^[*x*]_*R*_*i*__ is the set of equivalence classes produced by ∩_*i*=1_^*n*^ *R*_*i*_, we further have that ∩_*i*=1_^*n*^ *R*_*i*_ is coarser than *R*.

Then, choose arbitrarily $x\in \underline{\cap_{i=1}^{n}R_{i}}(X)$, and then we have [*x*]_∩_*i*=1_^*n*^*R*_*i*__⊆*X*, since [*x*]_∩_*i*=1_^*n*^*R*_*i*__⊇[*x*]_*R*_, we further obtain [*x*]_*R*_⊆*X*, implying $x\in \underline{R}(X)$, which together with $\underline{\Sigma_{i=1}^{n}R_{i}}(X)=\underline{R}(X)$ shows that $x\in \underline{\Sigma_{i=1}^{n}R_{i}}(X)$. And hence, $\underline{\cap_{i=1}^{n}R_{i}}(X)\subseteq \underline{\Sigma_{i=1}^{n}R_{i}}(X)$. Conversely, choose arbitrarily $x\in \underline{\Sigma_{i=1}^{n}R_{i}}(X)$, and by definition, there exists some equivalence relation *R*_*i*_ such that [*x*]_*R*_*i*__⊆*X*; since [*x*]_∩_*i*=1_^*n*^*R*_*i*__⊆[*x*]_*R*_*i*__, we further have [*x*]_∩_*i*=1_^*n*^*R*_*i*__⊆*X*, and thus $x\in \underline{\cap_{i=1}^{n}R_{i}}(X)$. Then $\underline{\Sigma_{i=1}^{n}R_{i}}(X)\subseteq \underline{\cap_{i=1}^{n}R_{i}}(X)$ holds owing to the arbitrariness of *x*. And hence, ∀*X*⊆*U*, $\underline{\Sigma_{i=1}^{n}R_{i}}(X)=\underline{\cap_{i=1}^{n}R_{i}}(X)$.

“⇐” It follows immediately by taking *R* = ∩_*i*=1_^*n*^ *R*_*i*_.

**Proposition 15 .**

Let (*U*, {*R*_*i*_}_*i*=1_^*n*^) be a multigranulation approximation space. Then (*U*, {*R*_*i*_}_*i*=1_^*n*^) is equivalent to a single-granular approximation space (*U*, *R*) if and only if ∀*X*⊆*U*, $\bar{\Sigma_{i=1}^{n}R_{i}}(X)=\bar{\cap_{i=1}^{n}R_{i}}(X)$.

**Proof**

It can be proved in a similar way as that in Proposition 14.

Main results in the present paper are summarized as follows.

**Proposition 16 .**

Let (*U*, {*R*_*i*_}_*i*=1_^*n*^) be a multigranulation approximation space. Then the following statements are equivalent:

- (*U*, {*R*_*i*_}_*i*=1_^*n*^) is equivalent to a single-granular approximation space (*U*, *R*),
- $\underline{H}$ forms a topology on *U*,
- $\bar{H}$ forms a topology on *U*,
- $\left(\bar{\Sigma_{i=1}^{n}R_{i}},\underline{\Sigma_{i=1}^{n}R_{i}}\right)$ forms an order-preserving Galois connection,
- $\bar{H}$ forms an union structure,
- $\underline{H}$ forms an union structure,
- ∀*X*⊆*U*, $\underline{\Sigma_{i=1}^{n}R_{i}}(X)=\underline{\cap_{i=1}^{n}R_{i}}(X)$,
- ∀*X*⊆*U*, $\bar{\Sigma_{i=1}^{n}R_{i}}(X)=\bar{\cap_{i=1}^{n}R_{i}}(X)$.

## 4. Concluding Remarks

In this paper, we consider the equivalence between multigranulation rough sets and single-granulation rough sets from the lattice-theoretic viewpoint. The obtained results will help us gain more insights into the mathematical structure of multigranulation rough sets. Along this research line, some interesting topics are worthy of further research; for instance, what is the structure of multigranulation rough sets induced by general binary relations or fuzzy relations on the universe and what is the connection between multigranulation rough sets and knowledge reasoning for multiple agents? We will report them in forthcoming papers.
